# Supplementary material for: Splice-Junction-Based Mapping of Alternative Isoforms in the Human Proteome
Source: Cell Rep. Author manuscript; Available in PMC 2020 Jan 15. (PMC6961840; doi:10.1016/j.celrep.2019.11.026)
Supplement: 2 [file NIHMS1546469-supplement-2.zip › DF1/PXD009737/TestisTrypsin/TestisTrypsin_76_PDPR_SAASQNAIVDADGR.pdf]

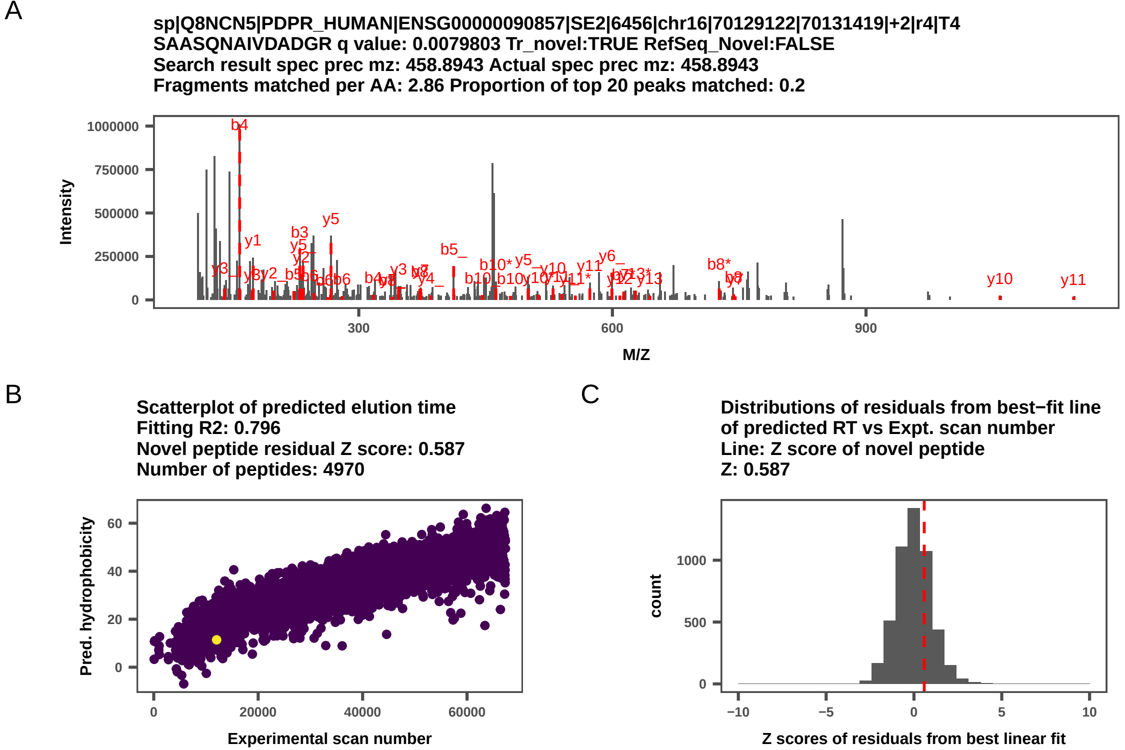
A
sp|Q8NCN5|PDPR_HUMAN|ENSG00000090857|SE2|6456|chr16|70129122|70131419|+2|r4|T4
SAASQNAIVDADGR q value: 0.0079803 Tr_novel:TRUE RefSeq_Novel:FALSE
Search result spec prec mz: 458.8943 Actual spec prec mz: 458.8943
Fragments matched per AA: 2.86 Proportion of top 20 peaks matched: 0.2
Intensity
1000000
750000
500000
250000
0
300
600
900
M/Z
b4
y1
b3
y5
y10
y11
B
Scatterplot of predicted elution time
Fitting R2: 0.796
Novel peptide residual Z score: 0.587
Number of peptides: 4970
Pred. hydrophobicity
60
40
20
0
0
20000
40000
60000
Experimental scan number
C
Distributions of residuals from best−fit line
of predicted RT vs Expt. scan number
Line: Z score of novel peptide
Z: 0.587
count
1000
500
0
−10
−5
0
5
10
Z scores of residuals from best linear fit
